# Chidamide with PEL regimen (prednisone, etoposide, lenalidomide) for elderly or frail patients with relapsed/refractory diffuse large *B*‐Cell lymphoma ‐results of a single center, retrospective cohort in China

**DOI:** 10.1002/hon.2979

**Published:** 2022-02-23

**Authors:** Yawen Wang, Hongwei Xue, Wei Song, Shuxin Xiao, Fanjing Jing, Tieying Dong, Lili Wang

**Affiliations:** ^1^ Department of Hematology The Affiliated Hospital of Qingdao University Qingdao China; ^2^ Department of Pathology The Affiliated Hospital of Qingdao University Qingdao China

**Keywords:** chidamide, histone deacetylase inhibitor, refractory/relapsed diffuse large *B*‐cell lymphoma, retrospective study

## Abstract

Treatment for relapsed/refractory Diffuse Large *B*‐Cell Lymphoma (R/R DLBCL) is evolving rapidly due to the emergence of novel drugs, of which histone deacetylase inhibitors (HDACis) are an important example. This study showed efficacy in patients with R/R DLBCL after failure of conventional therapies. We conducted a single‐center, retrospective study of 34 frail or elderly R/R DLBCL patients who had been treated off‐label with chidamide‐containing regimens from 2018 to 2020. *X* ^*2*^ or Fisher test were used to compare response rate and Kaplan‐Meier method was used to perform the survival analyses which compared with log‐rank test between different groups. The test standard was *p* < 0.05. In total, 34 patients with R/R DLBCL received CPEL+/‐R for at least 1 cycle were included. Most of them were refractory patients (*n* = 28,82.4%). The interim objective response rate (ORR) was 73.5% (32.4% complete remission [CR]), and the ultimate ORR was 50.0% (35.3% CR). After a median follow‐up of 13.1 months, the median progression‐free survival (PFS) was 10.5 months (95%CI 6.4–14.6) and the median overall survival (OS) was 19.3 months (95%CI 11.8–26.9). The 1 year expected PFS and OS rate was 43.0% and 73.7%, respectively. The most common grade 3/4 hematologic adverse events (AEs) were neutropenia (*n* = 11,32.3%) and anemia (*n* = 4, 11.8%) 0.23.5% (8/34) of all patients experienced grade 3/4 nonhematologic AEs. No treatment‐related deaths were observed. The study showed chidamide‐included regimen could be an option for R/R DLBCL patients ineligible for intensive chemotherapies. Current data showed favorable efficiency and moderate safety profile. Further study is warranted for better illustration of efficacy and usage in combination therapies.

## 1 INTRODUCTION

Diffuse large *B*‐cell lymphoma (DLBCL) is the most common non‐Hodgkin's lymphoma in adults and is biologically aggressive.^1^ The 5 year disease‐free survival (DFS) rate is ∼60% when patients are treated with rituximab, cyclophosphamide, doxorubicin, vincristine and prednisolone (R‐CHOP).^2^ Unfortunately, after an initial response to first‐line treatment, one‐third of patients relapse or experience refractory disease and require subsequent treatments,^3^, ^4^ of whom outcomes are generally poor, particularly for patients who are ineligible for intensive chemotherapy regimens and subsequent transplant treatment due to their advanced age, severe comorbidities, or unwillingness to receive intensive treatment.^5^, ^6^ Although there are lots of new drugs emerging now, the overall response rate (ORR) of refractory/relapsed DLBCL (R/R DLBCL) retains generally below 50.0% with a short survival time.^7^ In 2013 Mounier N et al reported that rituximab plus gemcitabine and oxaliplatin (R‐GemOx) regimen was effective for these ineligible patients with a 65% ORR, while the 5 year overall survival (OS) and progression‐free survival (PFS) rates were 13.9% and 12.8%, respectively.^8^ Moreover, patients with primary refractory disease or who experience relapse within 12 months after the first‐line treatment have a dismal outcome with a median OS of approximately 5–6 months.^9^, ^10^, ^11^ Therefore, there is a substantial unmet need for novel treatments for R/R DLBCL. Exploration of a therapy with acceptable toxicities as well as maximal output may be a pivotal point for these group of patients.

Chidamide is a novel orally active benzamide‐type histone deacetylase inhibitor (HDACi) that can induce growth arrest and apoptosis in blood and lymphoid‐derived tumor cells.^12^ In a phase II study, 79 relapsed/refractory peripheral *T*‐cell lymphoma (R/R PTCL) patients treated by chidamide achieved a 35% ORR (14% complete remission[CR]). Chidamide thus has been approved by the China Food and Drug Administration (CFDA) as a treatment for R/R PTCL.^13^ Pasqualucci et al reported that CREBBP/EP300 mutations were a major pathogenetic mechanism in most *B*‐cell non‐Hodgkin's lymphomas, which indicated that the use of drugs targeting acetylation/deacetylation mechanisms is a promising strategy.^14^ Recently, several kinds of HDACis were evaluated in clinical trials for *B*‐cell non‐Hodgkin's lymphoma (NHL). In a phase 2 study, mocetinostat, an isotype‐selective HDACi, achieved an 18.9% ORR in 41 R/R DLBCL patients, and nearly 1/3 got a stable disease state.^15^

Consequently, our single‐center retrospective analysis evaluated the efficacy and toxicity of chidamide plus prednisone, etoposide and lenalidomide with or without rituximab (CPEL+/‐R) for elderly and frail R/R DLBCL patients over a 2 year period.

## 2 PATIENTS AND METHODS

### 2.1 Patients

We conducted a retrospective, single‐center, off‐label, cohort study of all adult patients who had received chidamide for R/R DLBCL at the Affiliated Hospital of Qingdao University, China from 1 June 2018 to 1 June 2020 with the clinical cut‐off date of 31 July 2021. The patients were identified from an internal clinical database. The eligible patients were aged ≥18 years with a histologically confirmed diagnosis of CD20‐positive DLBCL based on the World Health Organization classification.^16^ Patient baseline characteristics included age, sex, Eastern Cooperative Oncology Group (ECOG) performance status, B symptoms, hematology and blood chemistry (CBC, differential; LDH, *β*2‐MG), Ann Arbor stage, extra‐nodal organ involvement, bone marrow involvement and International Prognosis Index (IPI) at the time of relapse or progression. Additionally, details on prior therapy were collected, including the number of treatment lines, best response, response duration of first‐line treatment, rituximab use, chimeric antigen receptor *T*‐cell (CAR‐T) therapy and autologous stem cell transplantation (ASCT) treatment status. Radiotherapy was not considered as a line of prior treatment. Data were obtained through a retrospective review of the electronic medical records.

This study was approved by the ethics committee of the Affiliated Hospital of Qingdao University and was carried out according to the guidelines of the Declaration of Helsinki. Chidamide was used as an off‐label compassionate treatment, and informed consent was obtained from all patients included in this study.

### 2.2 Treatment

Patients were treated on a 21–day cycle basis. Treatment schedule was as follows: chidamide, 20 mg, twice per week (biw), p.o.; prednisone 100 mg, p.o. QD on day 1–5 of each cycle; etoposide, 100 mg, p.o. QD on day 1–5; lenalidomide, 10 mg, p.o. QD on day 1–14. Patients who are financially affordable and willing to take on rituximab received rituximab at a dose of 375 mg/m^2^, iv, on day 1 on a 3 weeks basis.

### 2.3 Definition and criteria

Refractory disease was defined as stable disease (SD) or progressive disease (PD) in response to the immediate prior treatment and disease that relapsed within 12 months following a previously documented response (PR or CR), according to SCHOLAR‐1 study.^9^ Response to therapy was evaluated using the Lugano response criteria by Cheson 2014.^17^ Clinical response would be assessed every 2 cycles of regimen and we defined assessment after 4 and 8 cycles as interim and final response, respectively. For patients who experienced SD or PD and refused further treatment, evaluation after 2 or 3 cycle was included as interim and final response. Toxicity was reported according to the National Cancer Institute's Common Criteria for Adverse Events 4.0.

The primary objective of the study was to evaluate the activity of CPEL+/‐R in terms of the ORR and CR rate. The secondary objective was to evaluate the safety of CPEL+/‐R in terms of adverse events (AEs) and survival outcomes in terms of PFS and OS.

### 2.4 Statistics methods

PFS was calculated as the time from the first dose of CPEL+/‐R to the first documentation of disease progression or death. OS was calculated from the start of CPEL+/‐R treatment to the date of last follow‐up or death from any cause. Statistical analysis was performed using the software package IBM SPSS (v.19.0; IBM Corp). Measurement data with non‐normal distribution was expressed as median (range), while enumeration data was expressed as number of cases (%) and tested by *X* ^*2*^ method or Fisher exact test. Survival analysis were performed using the Kaplan‐Meier curves and log‐rank tests were performed to compare the pairs of subgroups in regard to the PFS and OS rate. Statistical significance was set at *p* < 0.05.

## 3 RESULTS

### 3.1 Patient characteristics

Thirty‐four patients with R/R DLBCL treated by CPEL+/‐R between 1 June 2018 and 1 June 2020 at our hospital were included in the study. Their clinical characteristic features are listed in Table 1. The median age of treatment start was 66 years (rang 38–84). Most patients (*n* = 31,91.2%) had advanced‐stage disease of Ann Arbor Stage III‐IV. Twenty five (73.5%) were documented with high or high‐intermediate IPI risk scores. Twenty‐three patients (67.7%) had poor functional status (ECOG score ≥2). Among all patients, 4 patients had a histology with transformed DLBCL. Two were transformed from mucosal‐associated marginal zone *B*‐cell lymphoma (MALT lymphoma), one from follicular lymphoma (FL) and one from mantle cell lymphoma (MCL). The median number of previous systemic therapies was 1 (rang, 1–3). Prior treatments of these patients included R‐CHOP, R‐GemOx, etoposide, methylprednisolone, high‐dose cytarabine, cisplatin (ESHAP); rituximab plus etoposide, prednisone, vincristine, cyclophosphamide, doxorubicin (R‐EPOCH); rituximab plus hyper fractionated cyclophosphamide, vincristine, doxorubicin, and dexamethasone alternating with high‐dose methotrexate and cytarabine (R‐hyper‐CVAD/MA). Two patients (5.9%) had ASCT and 1(2.9%) patient had CAR‐T cell treatment before the CPEL+/‐R regimen. 28 out of 34 patients were regarded as refractory, including 19 patients (55.9%) with relapse within 12 months and 9(26.5%) with no response to the previous chemotherapies.

**TABLE 1:** Baseline characteristics of patients before CPEL+/‐R regimen

	*N* = 34	%
Age at treatment start, *y*		
Median	66	
Range	38–84	
Age ≥65	21	61.8
Gender		
Male	19	55.9
Female	15	44.1
ECOG performance status		
0	2	5.9
1	9	26.5
≥2	2	67.7
	3	
B Symptoms		
Yes	1	44.1
	5	
No	1	55.9
	9	
Ann arbor stage		
II	3	8.8
III	2	5.9
IV	29	85.3
Elevated LDH	24	70.6
Elevated *β*2‐MG	12	35.3
IPI risk		
Low	3	8.8
Low‐intermediate	6	17.6
High‐intermediate	10	29.4
High	15	44.1
Bone marrow involvement	6	17.6
Exranodal involvement	12	35.3
Bulky disease^a^	15	44.1
Histology		
Non‐GCB	21	61.8
GCB	9	26.5
Transformed DLBCL	4	11.7
Response to last therapy		
No response	9	26.5
Relapse within 12 months	19	55.9
Relapse after 12 months	6	17.6
≥2 lines of previous chemotherapy	9	26.5

Abbreviations: *β*2‐MG, *β*2‐microglobulin; DLBCL, diffuse large *B*‐cell lymphoma; ECOG, Eastern Cooperative Oncology Group; GC, germinal center; IPI, Inter‐national Prognostic Index; LDH, lactate dehydrogenase.

^a^ Bulky disease is defined as that the maximum tumor diameter is larger than 10 cm according to MInT study.

All patients underwent CPEL+/‐R for at least 2 cycles. The median cycle of chidamide per patient was 4. Half of the patients (*n* = 17, 50.0%) were treated by rituximab (CPEL+R), and 10 of them received bendamustine + rituximab (BR) as a consolidation therapy after 4 cycles of CPEL+R. The dosage of chidamide was reduced from 20 mg biw to 15 mg or 10 mg biw for 8 patients (23.5%) because of severe AEs (including pruritus, neutropenia, impaired renal function et al.) as per physician's discretion. The distribution of chidamide‐included regimen and outcome of patients were shown in Table 2.

**TABLE 2:** Patient list of chidamide‐included regimen and outcome

Pt	Age, y	Ann arbor	Previous treatment	Regimen	No. of treatment cycle	Interim response	Final response	OS, month	PFS, month	Follow up
		stage								
1	56	IV A	R‐CHOP, R‐GemOx, CAR‐T	CPEL	8	PR	PR	28.9	28.9	Alive from the last follow‐up with no disease progression
2	55	IV B	R‐CHOP	R‐CPEL+BR	8	PR	CR	13.6	12.3	Disease progression after 8 months end of treatment and alive from the last follow‐up
3	72	IV A	R‐CDOP	CPEL	4	PR	PR	31.3	31.3	Chidamied discontinued as renal disfunction and alive from the last follow‐up with no disease progression
4	58	III A	R‐CHOP	R‐CPEL	6	SD	PD	4.8	4.2	Discontinue treatment after PD, alive with supportive care from the last follow up
5	71	IV A	R‐CDOP+ intrathecal MTX	CPEL	2	SD	SD	1.5	1.5	Died of disease progression
6	66	IV B	CHOPE, R‐CDOP, lenalidomide	CPEL	8	PR	PD	12.9	8.1	Chidamide dose reduction to 20 mg biw due to pruritus, died of disease progression
7	79	IV B	R‐miniCHOP	CPEL	8	CR	CR	20.3	18.3	Died of disease progression
8	56	IV A	R‐EPOCH	R‐CPEL	2	SD	SD	13.3	2.2	Continued with CART after 2 months end of treatment, disease free of last follow up
9	66	III A	R‐CDOP	R‐CPEL+BR	8	CR	CR	24.5	21.3	Disease progressed after 14 months end of treatment and alive from the last follow‐up with no disease progression
10	73	IV A	R‐CDOP	CPEL	6	SD	PD	4.5	3.0	Died of disease progression
11	74	II A	R‐CDOP	R‐CPEL	8	CR	CR	8.9	8.9	Alive from the last follow‐up with no disease progression
12	65	IV B	CHOP+RT	CPEL	8	PR	PR	17.8	13.4	Died of disease progression
13	57	IV A	R‐CDOP, EPOCH, ASCT	CPEL	2	PD	PD	3.4	2.3	Died of disease progression
14	68	III A	R‐CDOP	CPEL	8	CR	PD	10.7	8.3	Died of disease progression
15	41	IV B	R‐EPOCH	R‐CPEL	4	PD	PD	10.6	4.9	Disease progression after 4^th^ cycles of CPEL, resume ASCT and alive from the last follow‐up
16	58	Missing	CHOP, R‐COP, ESHAP	CPEL	8	PR	PD	19.3	10.5	Disease progression after 6^th^ cycles of CPEL, resume RCHOP; died of disease progression xx month after end of CPEL.
17	62	IV A	R‐HyperCVAD/R‐MA	R‐CPEL	6	CR	CR	34.7	34.7	Alive from the last follow‐up with no disease progression
18	75	IV A	R‐CHOP	R‐CPEL+BR	8	CR	CR	18.3	18.3	Alive from the last follow‐up with no disease progression
19	74	II B	R‐CHOP	CPEL	6	CR	PD	12.2	11.1	Disease progression after the 6^th^ cycles of CPEL and died of disease progression
20	61	IV A	R‐CHOP, GemOx	CPEL	6	CR	CR	28.9	28.9	Alive from the last follow‐up with no disease progression
21	84	IV A	RC2PET	CPEL	5	PR	PD	7.8	4.4	Died of disease progression
22	61	IV B	R‐CDOP, ASCT	R‐CPEL+BR	8	PR	PD	9.2	7.2	Disease progression after 2 months of end BR regimen treatment
23	76	IV A	R‐CDOP	R‐CPEL+BR	8	CR	CR	14.9	14.9	Alive from the last follow‐up with no disease progression
24	65	IV B	R‐CHOP	R‐CPEL	3	PD	PD	4.8	1.7	Died of disease progression
25	75	IIE A	R‐CDOP	R‐CPEL+BR	6	SD	SD	9.9	9.9	Discontinuation of treatment as skin infections and alive from the last follow‐up with no disease progression
26	68	IV B	R‐CDOP	R‐CPEL+BR	8	PR	PR	10.9	10.9	Alive from the last follow‐up with no disease progression
27	61	IV B	R‐EPOCH	CPEL	6	CR	CR	20.9	5.7	Disease progression 2 months after end of 4 cycles of CPEL, undergone RDGP and CART; CR from last follow‐up
28	77	IV A	R‐miniCDOP+RT	CPEL	8	PR	CR	23.7	16.1	Disease progression after 10 months end of CPEL, resume BR and got CT, alive from the last follow‐up
29	55	III A	R‐EPOCH	R‐CPEL+BR	8	PR	PR	13.5	13.5	Alive from the last follow‐up with no disease progression
30	67	IV A	CHOP, R‐CHOPE	CPEL	6	PR	PD	20.2	18.3	Disease progression after the 7^th^ cycle of CPEL and died of PD
31	55	IV A	R‐CDOP	CPEL	8	PR	PD	14.2	9.8	Disease progression after the 7^th^ cycle of CPEL, undergone RGemOX, died of PD
32	38	IV B	R‐EPOCH	R‐CPEL+BR	8	PR	CR	8.9	8.9	Alive from the last follow‐up with no disease progression
33	68	IV A	CHOP, R‐GDP+lenalidomide	R‐CPEL	4	SD	PD	9.7	5.0	PD after 3 cycles of R‐CPEL, underwent BR+ibutirib and achieved PR; died from PD
34	70	III A	R‐CDOP	R‐CPEL+BR	8	CR	CR	11.7	11.7	Alive from the last follow‐up with no disease progression

Abbreviations: ASCT, autologous stem cell transplantation; CAR‐T, chimeric antigen receptor *T*‐cell treatment; CPEL, chidamide plus prednisone etoposide and lenalidomide; CR, compldiete response; ESHAP, etoposide, methylprednisolone high‐dose cytarabine, cisplatin; PD, progressive disease; PR, partial response; Pt, patient no; R‐C2PET, rituximab cyclophosphamide, etoposide thalidomide; R‐CDOP, rituximab cyclophosphamide, liposome adriamycin vincristine, prednisone; R‐CHOP, rituximab, cyclophosphamide doxorubicin, vincristine prednisone; R‐CPEL, chidamide plus prednisone etoposide and lenalidomide, with rituximab; R‐CPEL+BR, chidamide plus prednisone etoposide and lenalidomide with rituximab follow with bendamustine plus rituximab as consolidation; R‐GDP, gemcitabine cisplatin, dexamethasone; R‐GemOX, rituximab, gemcitabine and oxaliplatin; R‐Hyper CVAD/R‐MA: rituximab plus hyper fractionated cyclophosphamide vincristine, doxorubicin and dexamethasone alternating with high‐dose methotrexate and cytarabine; SD, stable disease.

### 3.2 Response

All patients were evaluable for response. In an intention‐to‐treat analysis 11 patients (32.4%) achieved CR and 14 patients (41.2%) showed PR, resulting in an interim ORR of 73.5% after 4 cycles of CPEL+/‐R regimen. However, as 11 patients showed disease progression during the 5th–8th cycles, the terminal ORR was 50.0% (35.3% CR) at 1 month after completing 8 cycles of the CPEL+/‐R regimen (Table 3).

**TABLE 3:** Response and survival outcomes of all patients

Response	Interim		Final	
	*N* = 34		*N* = 34	
	*N*	%	*N*	%
CR	11	32.4	12	35.3
PR	14	41.2	5	14.7
SD	6	17.6	3	8.8
PD	3	8.8	14	42.1
ORR	25	73.5	17	50.0

**FIGURE 1:**
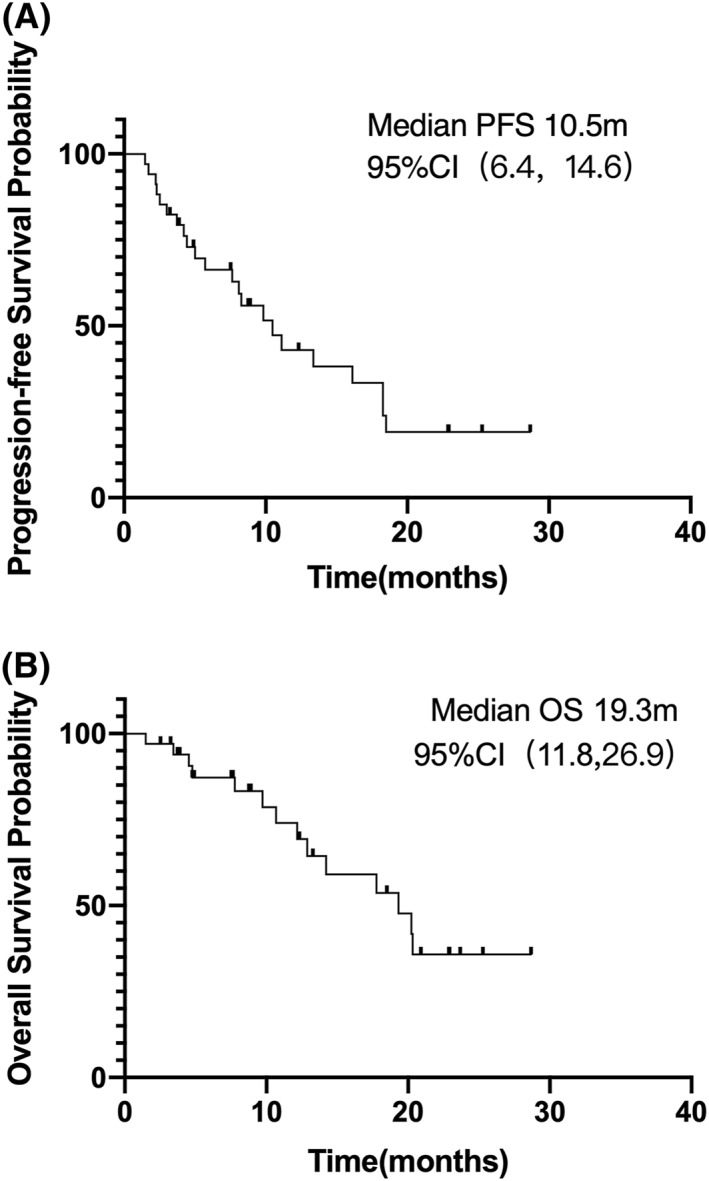
Progression‐free survival (PFS) and overall survival (OS) of 34 patients treated by Chidamide with PEL regimen (prednisone, etoposide, lenalidomide). (A), PFS of all patients (*n* = 34) with median PFS of 10.5 months. (B), OS of all patients (*n* = 34) with median OS of 19.3 months

The median follow‐up of these patients was13.1 months. 20 patients (58.8%) were alive at the time of CCOD. The median PFS was 10.5 months (95% CI 6.4–14.6), and the median OS was 19.3 months (95% CI 11.8–26.9). The 1 year expected PFS rate was 43.0% and the 1 year expected OS rate was 74.0% (Figure 1).

**FIGURE 2:**
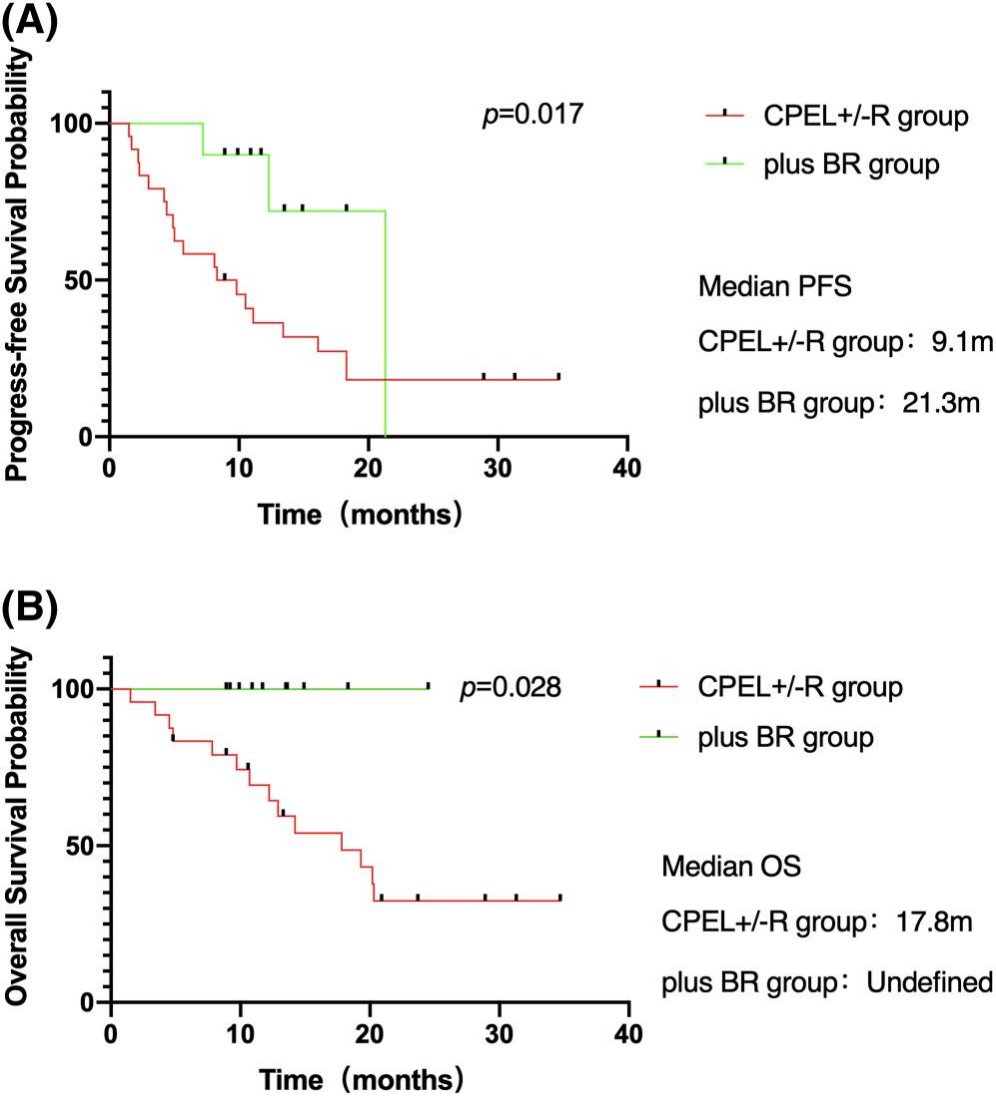
Probability of progression‐free survival (PFS) and overall survival (OS) of patients, compare by regimen. (A), Comparison of probability of PFS of patients between CPEL (*n* = 24) and CPEL plus bendamustine + rituximab (BR) consolidation group (*n* = 10). *p* = 0.017; (B) Comparison of OS of patients between CPEL (*n* = 24) and CPEL plus BR consolidation group (*n* = 10). *p* *=* 0.028

**FIGURE 3:**
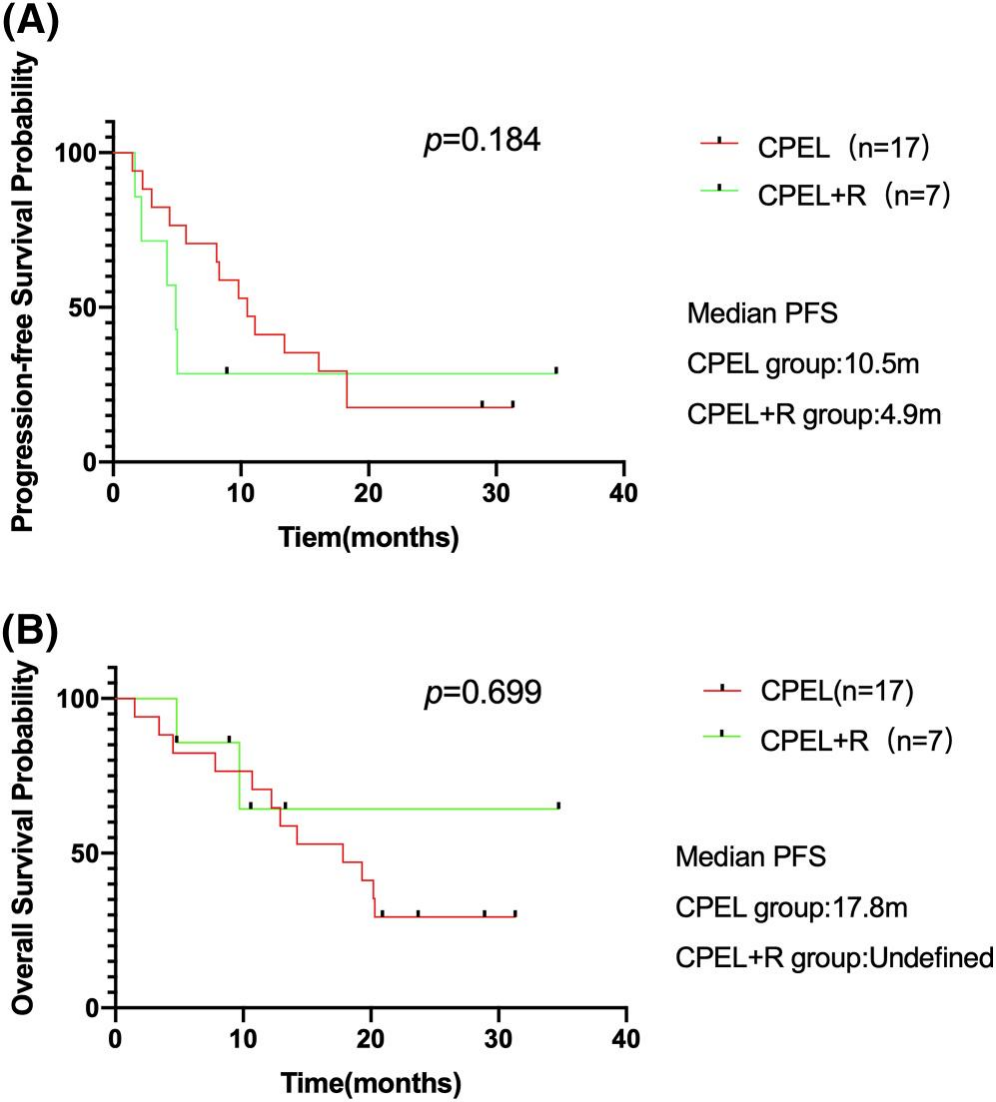
Comparison of Probability of progression‐free survival (PFS) and overall survival (OS) between groups of patients receiving CPEL (*n* = 17) versus CPEL+R (*n* = 7). (A), Progression‐free survival for patients of the two regimen group with the median PFS being 10.5 months of the CPEL group and 4.9 months CPEL+R group, *p* = 0.184 (B) OS comparison of the two groups, with median OS being 17.8 months for the CPEL group and undefined for the CPEL+R group, *p* = 0.699

Patients who received 4 cycles of BR regimen as consolidation therapy after 4 cycles CPEL+/‐R (plus BR group) had a better PFS (*p* = 0.017) and OS (*p* = 0.028) than those received 8 cycles of CPEL+/‐R (Figure 2). Plus BR group had a better final ORR than the CPEL+/‐R group (80.0% vs. 37.5%, *p* = 0.029), while there was no statistical difference in terms of final CR rate (60.0% vs. 25.0%, *p* = 0.402). There was no survival difference between the CPEL regimen treated group (*n* = 17,50.0%) and CPEL+R regimen group (*n* = 7,20.6%) (Figure 3).

**FIGURE 4:**
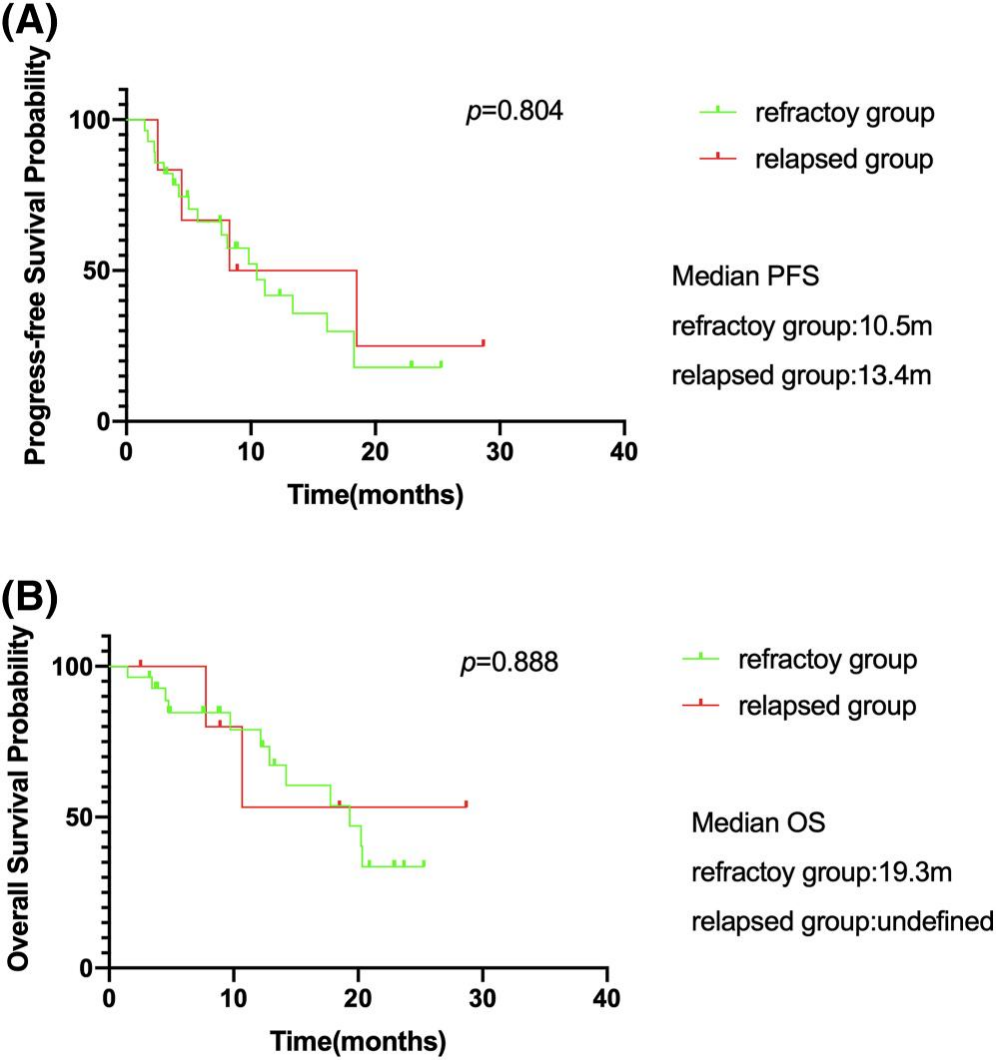
Progression‐free survival (PFS) and overall survival (OS) for refractory (*n* = 28) versus relapsed group (*n* = 6) of Diffuse large *B*‐cell lymphoma (DLBCL) patients. (A) Median PFS of refractory DLBCL patients is 10.5 and 13.4 months for relapsed group, *p* = 0.804; (B) Median OS of refractory DLBCL patients is 19.3 months and undefined for relapsed group, *p* = 0.888

There were 28 refractory patients (20 patients received CPEL+/‐R regimen and 8 patients treated with R‐CPEL followed with BR regimen), which achieved an ORR with 67.9% (25.0% CR) at the interim point and 50.0% (32.1% CR) at the terminal point as 8 patients progressed during the 5th–8th cycles. While the relapsed group (*n* = 6,17.4%), 4 patients treated by CPEL+/‐R regimen and 2 received R‐CPEL plus BR regimen, achieved an ORR with 100.0% (66.7% CR) and 50.0% ( 50.0% CR) at the interim and terminal point, respectively. Chidamide‐included regimen had similar response rates in the refractory and relapsed groups (*p* = 0.132 and 0.07 for interim ORR and CR rate, respectively; *p* = 0.672 and 0.351 for terminal ORR and CR rate, respectively), as well as in survival (*p* = 0.888 and 0804 for OS and PFS, respectively)(Figure 4).

The regimen containing BR as a consolidated therapy produced better overall survival and progression‐free survival rates and the difference was statistically significant by the means of the K‐M log‐rank test, but appeared to have no significant difference by means of the Cox regression analysis (details not list).

### 3.3 Toxicity

Sixty‐nine adverse events (AEs) were recorded in 29 patients (85.3%) and one event were serious AE. The SAE was grade 4 pulmonary embolism (PE) reported in a 74 year old female patient and caused drug delay. 25 AEs were grade 3 or 4 and no grade 5 AEs. Eight AEs (including pruritus, neutropenia, impaired renal function et al.) leaded to drug reduction and 2 AEs resulted in treatment discontinuation(including impaired renal function and skin infection). The most common grade 3/4 hematologic AEs were neutropenia (*n* = 11,32.3%) and anemia (*n* = 4,11.8%). Eight patients (23.5%) experienced grade 3/4 non‐hematologic AEs. The common non‐hematologic AEs were fatigue, infection, thrombogenesis, anorexia and hyponatremia, which alleviated with symptomatic supportive treatment. No treatment‐related deaths were observed.

## 4 DISCUSSION

To our knowledge, the present study included the largest cohort of subjects with R/R DLBCL treated with chidamide without a clinical trial. Our study indicated that the CPEL+/‐R regimen can be an efficient and safe choice for R/R DLBCL patients who were ineligible for intensive chemotherapy.

Treatment of R/R DLBCL are especially challenging given its aggressive nature, low response to chemotherapy and deteriorated condition for many late stage and elderly patients. As reported in an international multicohort retrospective NHL research study (SCHOLAR‐1),^9^ for patients with refractory DLBCL the objective response rate was 26.0% to the second or later line of therapy and the median OS was 6.3 months. In our study 61.8% of the patients were old‐age (≥65), the majority (*n* = 28,82.4%) had refractory disease and 73.5% were in high IPI risk groups. The ORR in these unfit patients reached 73.5% (with 32.4% CR) at the interim point of CPEL+/‐R therapy and 50% (35.3% CR) at final point. The 1 year expected PFS rate is 43.0% and OS rates 74.0%. Data of the study showed an encouraging overall efficacy of chidamide‐included regimens.

In the refractory group of patients (*n* = 28), ORR reached 67.9% (*n* = 19) after 4 cycles of CPEL+/‐R, suggesting a possible advantage of overcoming drug resistance of the regimen, not only against rituximab but also other antitumor agents. Previous studies indicated that acquired resistance to chemotherapy was related to changes in the tumor cell epigenome, which may directly interfere with the cell cycle and some key apoptosis regulators.^18^ HDAC inhibitors (with Chidamide being one of its oral formulation) have been shown to synergize and revert antitumor drug resistance when in combination with other chemotherapy drugs in studies and investigations. The loss of CD20/MCHII is a major obstacle for the retreatment of R/R DLBCL with rituximab‐associated regimens.^19^ Several HDAC inhibitors can reverse the unfavorable impact of the loss of MHCII expression^20^, and chidamide was reported to significantly enhance rituximab‐induced tumor growth inhibition in vitro and in vivo.^21^ Lenalidomide and HDACi possess overlapping identical immunomodulatory effects, which might produce synergy in anti‐tumor immunity by upregulating the expression of major histocompatibility complex class I and II proteins and costimulatory molecules.^22^ Several phase II clinical studies have evaluated the efficacy of combined HDACi and lenalidomide for aggressive non Hodgkin lymphoma. Hopfinger et al explored a regimen consisting of vorinostat, lendalidomide and dexamethasone in 8 R/R PTCL patient and one patient with CR and an ORR of 25% were reported.^23^ In 2020, Shaoxuan Hu et al^24^ reported successful treatment with lenalidomide plus chidamide combination therapy in 3 heavily treated patients with non‐Hodgkin lymphoma. Based on the above literature and data, it could be justifiable and promising to adopt chidamide in combination with other drugs to treat NHL patients in a refractory context.

Another advantage of the chidamide combination is allowance for decrease in chemotherapy doses with reduced toxicities and adverse effects. Among the 34 patients, only 6 patients (17.6%) had grade 4 neutropenia, 4(11.8%) had grade 3 anemia and 2(5.9%) had grade 3 thrombocytopenia in terms of hematological AEs. During the treatment, the patients took pills at home after 1 day of an intravenous administration of rituximab and visited their physician in outpatient. Reduction in hospitalization offers better cost‐effectiveness and convenience. In this study, two patients who progressed after a certain PFS from the CPEL regimen (PFS is 172 and 67 days, respectively) underwent CAR‐T treatment and achieved CR again with a long PFS, which is inspiring us a lot. Although CAR‐T therapy offer a promising ORR ranging from 52.0%–82.0% for the R/R DLBCL patients,^25^, ^26^, ^27^ achieving adequate disease control and remission periods that allow enough time to manufacture and process viable CAR products is an unmet clinical need. It suggested that CPEL+/‐R regimen may be act as a bridge to CAR‐T cell therapy for the frail patients. One limitation to our study is the small sample size of patients. Larger sample size and well‐designed clinical trials are needed before a clear conclusion can be drawn. Another limitation of the cohort is the heterogeneity of regimen used in patients. While such condition happens in the real‐world studies, where choices need to be made and adjusted on every single case's basis, these data do provide useful information and reference for physicians and investigators to design further study and regimen.

## 5 CONCLUSION

In summary, the study illustrated the efficacy and safety of the CPEL+/‐R regimen for R/R DLBCL patients who were ineligible for intensive chemotherapy and ASCT. Future studies are needed to define the optimal schedule.

## CONFLICT OF INTEREST

The authors declare that the research was conducted in the absence of any commercial or financial relationships that could be construed as a potential conflict of interest.

## AUTHOR CONTRIBUTIONS

Yawen Wang were responsible for data collection and interpretation, statistical analysis, literature research, and manuscript writing. Wei Song were responsible for data collection and patients' care. Shuxin Xiao, Fanjing Jing, Tieying Dong treated the patients and provided data. Lili Wang was responsible for pathological consultation. Hongwei Xue designed the study and approved the final version. All authors reviewed the manuscript.

### PEER REVIEW

The peer review history for this article is available at https://publons.com/publon/10.1002/hon.2979.

## Data Availability

The data that support the finding of this study are available from the corresponding author upon reasonable request.
